# Predicting Osteopathic Medical Students' Performance on the United States Medical Licensing Examination From Results of the Comprehensive Osteopathic Medical Licensing Examination

**DOI:** 10.7759/cureus.14288

**Published:** 2021-04-04

**Authors:** Travis Smith, Mark Kauffman, J. Bryan Carmody, James Gnarra

**Affiliations:** 1 Emergency Medicine, Lake Erie College of Osteopathic Medicine, Bradenton, USA; 2 Family Medicine, Lake Erie College of Osteopathic Medicine, Bradenton, USA; 3 Pediatrics, Eastern Virginia Medical School, Norfolk, USA; 4 Microbiology and Immunology, Lake Erie College of Osteopathic Medicine, Bradenton, USA

**Keywords:** usmle, comlex-usa, residency selection, match

## Abstract

Introduction

The reliance on the United States Medical Licensing Examination (USMLE) Step 1 scores in residency selection creates problems for osteopathic medical students and the programs that review their applications. Although many osteopathic students take the USMLE to improve their standing for residency selection, students who score poorly may harm their candidacy. Simultaneously, programs unfamiliar with the Comprehensive Osteopathic Medical Licensing Examination (COMLEX-USA) may struggle to evaluate applicants who have not taken USMLE.

Objective

To determine the association between COMLEX-USA Level 1 and USMLE Step 1 scores and derive an equation that could be used to predict USMLE performance or approximate USMLE scores for applicants who have only taken COMLEX-USA.

Methods

We reviewed COMLEX-USA Level 1 and USMLE Step 1 scores for all students at the Lake Erie College of Osteopathic Medicine (LECOM), Bradenton campus, from January 2012 until December 2016. Linear regression was used to evaluate the relationship between COMLEX-USA Level 1 and USMLE Step 1 scores.

Results

Overall, 2097 students took both examinations during the study period. Every one-point increase in COMLEX-USA was associated with a 0.15 point increase in USMLE Step 1 score (standard error 11.5; model R^2^ 0.56). On average, students scored 30 percentile points lower on USMLE Step 1 than on COMLEX-USA, and 24% of students scoring <500 on COMLEX-USA Level 1 failed USMLE Step 1.

Conclusions

Students or programs interested in predicting performance on USMLE Step 1 from performance on COMLEX-USA Level 1 can do so with the following equation: USMLE Step 1 = 0.15 (COMLEX-USA Level 1) + 138.7.

## Introduction

In recent years, a substantial and growing number of osteopathic medical students have taken the United States Medical Licensing Examination (USMLE). By 2018, approximately 60% of osteopathic medical students had taken the USMLE [1]. Although allopathic medical students are required to take the USMLE to pursue licensure, osteopathic students take the USMLE in the hopes of improving their standing in residency selection. Despite statements from professional organizations urging program directors to consider the USMLE and Comprehensive Osteopathic Medical Licensing Examination (COMLEX-USA) to be equivalent [2], many residency program directors continue to encourage osteopathic applicants to take the USMLE [3].

The reliance on USMLE Step 1 scores in residency selection can present a dilemma for osteopathic students and their advisors. Although a student who scores highly may improve his or her chances in residency selection, a student who scores poorly - or who fails the examination altogether - is unlikely to do so. Similarly, while a growing number of osteopathic medical schools now require their students to take the USMLE in addition to COMLEX-USA, the benefit of these policies must be weighed against the additional costs imposed on students and the likelihood that some students may have their residency applications harmed by poor performance.

Multiple studies have demonstrated an association between higher scores on USMLE Step 1 and COMLEX-USA Level 1 [4-9] However, many of these studies did not report all of the data necessary for a test-taker to predict his or her USMLE score interval [5,8,9]. Additionally, because the mean score for USMLE Step 1 has been increasing by approximately 1.2 points/year [10], older formulae significantly underpredict performance for contemporary test-takers [4,6].

Here, we sought to evaluate the relationship between scores on the COMLEX-USA Level 1 and USMLE Step 1 examinations in a large sample of osteopathic medical students; compare the relationship to previously published reports; and generate formulae to predict the most likely USMLE Step 1 score as well as the range of possible outcomes from a given COMLEX-USA Level 1 score.

## Materials and methods

Setting and Participants

We reviewed the COMLEX-USA Level 1 and USMLE Step 1 scores for all students at the Lake Erie College of Osteopathic Medicine (LECOM), Bradenton campus, who took both exams between January 1, 2012, and December 31, 2016. COMLEX-USA and USMLE scores were obtained from educational records held by the campus registrar and were deidentified prior to analysis. For students who took either exam more than once, performance on the first exam attempt was analyzed. Percentiles for COMLEX-USA and USMLE scores were determined using the reference standards provided by the testing bodies [11,12].

Statistical Analysis

Descriptive statistics (including median and interquartile ranges (IQRs) for performance on each test) were calculated for the study population. To evaluate the association between COMLEX-USA and USMLE scores, linear regression was performed, while logistic regression was used to evaluate the association between scores and examination failure. A two-sided significance level of 0.05 was set for all tests. All statistical analyses were performed using IBMⓇ SPSS version 26 (Armonk, NY).

IRB Statement

Because the investigators received only deidentified paired score data from the campus registrar, the LECOM Institutional Review Board (IRB) determined this research to be IRB-exempt and the need for consent to be waived.

## Results

From 2012 to 2016, there were 2097 students at LECOM who took both the COMLEX-USA Level 1 and the USMLE Step 1 examinations. The median COMLEX-USA Level 1 score was 596 (IQR: 543-650; range: 372-999). The median COMLEX-USA percentile was 80 (IQR: 61-93). In comparison, the median USMLE Step 1 score was 228 (IQR: 215-239, range: 162-268), with a median score percentile of 45 (IQR: 23-67).

**Figure 1:**
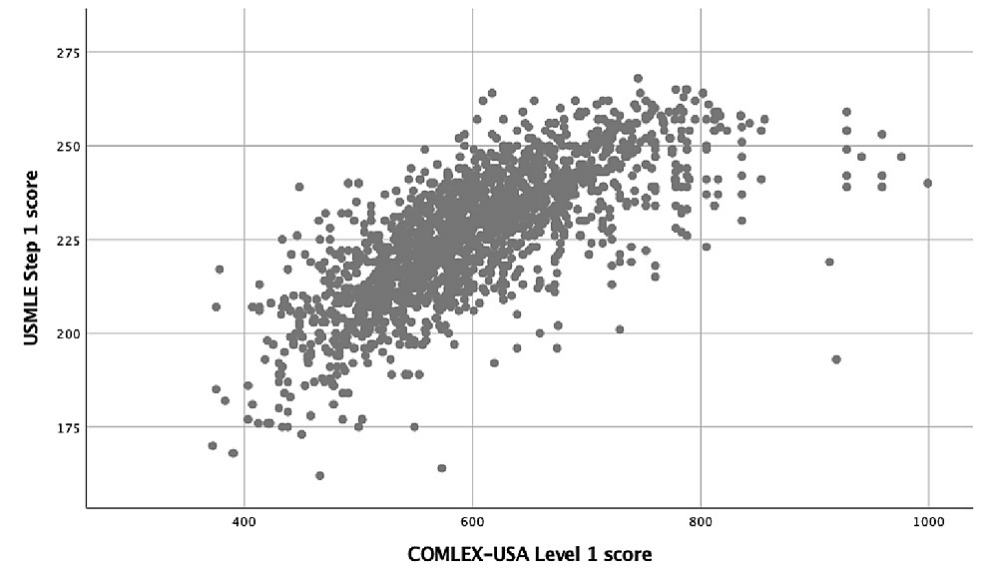
Scatterplot of individual student's performance on the COMLEX-USA Level 1 and USMLE Step 1 examinations. COMLEX-USA: Comprehensive Osteopathic Medical Licensing Examination; USMLE: United States Medical Licensing Examination.

Pairs of COMLEX-USA and USMLE scores are shown in the scatterplot in Figure 1. There was a linear association between COMLEX-USA Level 1 and USMLE Step 1 scores, with every 1 point increase in COMLEX-USA Level 1 associated with a 0.146 point increase in USMLE Step 1 score (95% CI: 0.140-0.152, *p* < 0.001). The USMLE Step 1 score could be estimated from the COMLEX-USA Level 1 score using the equation:

USMLE Step 1 = 0.15 (COMLEX-USA Level 1 score) + 138.7

For this model, the standard error of the estimate was 11.5, with model R^2^ = 0.56.

Similarly, there was a linear association between COMLEX-USA Level 1 percentiles and percentiles for USMLE Step 1 performance, with every 1 percentile increase in COMLEX-USA associated with a 0.84 percentile increase on USMLE Step 1 (95% CI: 0.81-0.87; *p* < 0.001). The USMLE Step 1 percentile could be estimated from the COMLEX-USA Level 1 percentile using the equation:

USMLE Step 1 percentile = 0.84 (COMLEX-USA Level 1 percentile) - 17.7.

For this model, the estimate's standard error was 17.4, with R^2^ = 0.54.

Only seven (0.3%) students who took both exams failed COMLEX-USA Level 1 (score < 400), while 69 (3.3%) failed USMLE Step 1 (score < 194). The risk of USMLE Step 1 failure varied by COMLEX-USA score. For instance, among students scoring < 500 on COMLEX-USA Level 1, 55/227 (24.2%) failed USMLE Step 1, while only 14/1870 (0.7%) of students with COMLEX-USA Level 1 > 500 failed USMLE Step 1 (odds ratio [OR]: 42.4; 95% CI: 23.1-77.8; *p* < 0.001).

**Figure 2:**
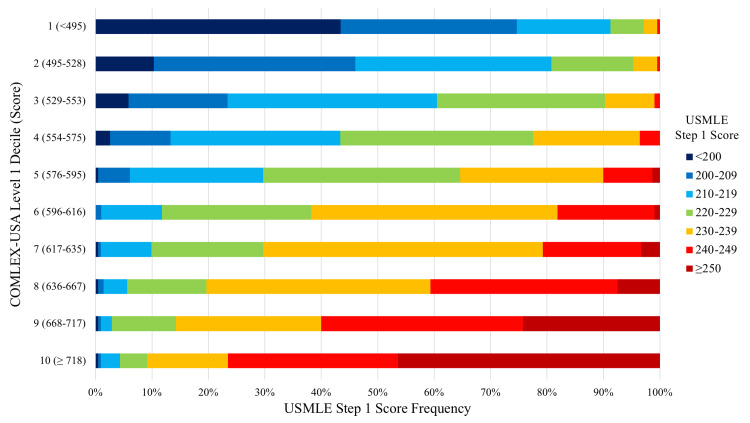
Frequency of USMLE Step 1 scores by decile of COMLEX-USA Level 1 performance. COMLEX-USA: Comprehensive Osteopathic Medical Licensing Examination; USMLE: United States Medical Licensing Examination.

Figure 2 demonstrates the range of observed USMLE Step 1 scores by decile of COMLEX-USA performance. For instance, among the 204 test-takers whose COMLEX-USA Level 1 score fell in the 6th decile of performance in our population (range: 596-616), 89 (43.6%) scored between 230-239, while just two (1%) test-takers scored <210 or >/= 250 on USMLE Step 1.

## Discussion

Here, we present the association between COMLEX-USA Level 1 and USMLE Step 1 scores in a large sample of 2097 students from a single osteopathic medical school from 2012 to 2016. There was a relatively strong linear relationship between performance on these exams, with variation in COMLEX-USA scores explaining approximately 56% of the variation in USMLE Step 1 performance, and every one-point increase in COMLEX-USA Level 1 score being associated with a 0.15 point increase in USMLE Step 1 score.

These findings should inform osteopathic medical students who are considering whether to take the USMLE, as well as their advisors. When osteopathic medical students choose to take a second licensing examination, they do so in the hope that this will benefit their residency application. However, this potential benefit comes with risk, as obtaining a low score or failing the examination altogether may adversely impact residency competitiveness. Although COMLEX-USA Level 1 and USMLE Step 1 scores are highly correlated, the CI is relatively wide. The data in Figure 2 may be especially useful to individual students in assessing the likelihood of receiving a USMLE score that will improve their residency application. Certain thresholds of COMLEX-USA performance may serve as useful guidelines. For instance, students scoring <600 on COMLEX-USA Level 1 had a median USMLE score of 216 (IQR: 201-226), and 20% of students scored 205 or lower. In comparison, students scoring >/= 600 had a median score of 238 (IQR: 231-247), and 20% of these students scored 249 or higher.

Similarly, these analyses may be beneficial to deans and administrators considering whether to require students to take the USMLE. On average, students who took both exams scored 30 percentile points lower on USMLE Step 1 than they did on COMLEX-USA Level 1. Students scoring <500 on COMLEX-USA Level 1 were particularly at risk for failing USMLE Step 1 (absolute risk: 24%; OR: 42.2). Schools at which a significant number of students have COMLEX-USA scores that fall below this threshold might reasonably choose not to require USMLE Step 1 for all students.

In our sample, only 3.3% of students failed USMLE Step 1, a figure that is lower than the 6-7% first-time failure rate for all first-time osteopathic test-takers during the time period of our study [13]. However, it is notable that approximately 85% of LECOM students who took COMLEX-USA Level 1 also took the USMLE Step 1 exam. Although there are many reasons why a student might decline to take both examinations (including the financial and opportunity costs of taking both exams, or the pursuit of residency training opportunities where USMLE scores are not necessary for full consideration), it seems likely that students who took only the COMLEX-USA Level 1 might have had a higher risk for failure on USMLE Step 1 than those who chose to take both examinations. Other studies have found that approximately 7% of students failed the USMLE Step 1 examination despite passing COMLEX-USA Level 1 [8].

These findings may also be of interest to residency program directors. Because there are differences in content between the COMLEX-USA Level 1 and USMLE Step 1 examinations, it is impossible to “convert” scores from one exam to another. Nonetheless, the pressure on osteopathic students to take the USMLE examinations provides empiric proof that there is a strong desire among many program directors to compare medical knowledge among all applicants. While the National Board of Osteopathic Medical Examiners publishes percentiles for COMLEX-USA performance [11], these do not allow programs to directly compare osteopathic applicants to applicants who took only the USMLE. In contrast, by using the regression equation derived from our dataset, program directors can obtain a point estimate of what an applicant’s USMLE Step 1 score might have been (USMLE Step 1 = 0.15 (COMLEX-USA Level 1) + 138.7).

Our analyses are not the first to use COMLEX-USA Level 1 scores to predict USMLE Step 1 scores, and it is useful to compare our results to those reported in three previous papers that included complete descriptions of their linear regression model [4,6,7]. In 2014, Lee et al. analyzed test scores for 1016 students at a single college of osteopathic medicine who took both COMLEX-USA Level 1 and USMLE Step 1 between 2006 and 2012 [7]. They reported the following linear regression model: USMLE Step 1 = (0.24 x COMLEX-USA Level 1) + 82.6). When this equation was used to predict USMLE Step 1 scores in our study, it generally performed well, with observed scores that differed from those predicted by a median of just 1 point. However, the Lee et al. equation substantially outperformed the older models by Slocum and Louder [4] and Sarkoet al. [6], both of which systematically underpredicted USMLE Step 1 scores in our population by 12-15 points on average.

In February 2020, the USMLE announced that results of the Step 1 examination would be reported as pass/fail beginning as soon as January 2022. Although this may obviate the need to counsel students on whether to take both licensing examinations, it will not negate the desire of program directors to have a common standard by which to evaluate applicants’ medical knowledge. Even after new Step 1 results are reported as pass/fail, programs will still likely encounter some applicants who apply with numeric scores obtained in previous years.

This work has many strengths. This is the largest study to date evaluating pairs of COMLEX-USA Level 1 and USMLE Step 1 scores, with more than twice as many data points as the largest previous work [7]. Even with the USMLE’s pass/fail announcement, this topic remains highly relevant to both undergraduate and graduate medical education, as the USMLE Step 1 score remains the single most commonly used metric when evaluating residency applicants to interview [14]. 

Nonetheless, this study has some important limitations. First, this is a single-center study. While we believe the study population to be typical for many osteopathic medical schools, our results may not be generalizable to students from schools that systematically differ from LECOM. Second, our analyses were limited to the evaluation of COMLEX-USA and USMLE scores, which were deidentified when obtained for analysis. Other factors that likely impact test performance - such as prior academic performance, amount of dedicated preparation, and study resources used - could not be assessed. Finally, any analysis that aims to predict standardized test performance will necessarily be limited by the inherent imprecision of these tests themselves. The USMLE Step 1 examination has a standard error of measurement of approximately six points [12], meaning that if an examinee were tested repeatedly using different sets of questions testing the same content, the 95% CI for the examinee’s score would span 24 points.

## Conclusions

There is a linear relationship between osteopathic medical student performance on the COMLEX-USA Level 1 and the USMLE Step 1 examinations, in which every 1 point increase in COMLEX-USA Level 1 is associated with a 0.15 point increase in USMLE Step 1 score. Those interested in predicting performance on USMLE Step 1 from an individual COMLEX-USA Level 1 score can do so with the following equation: USMLE Step 1 = 0.15 (COMLEX-USA Level 1) + 138.7. Before taking a non-mandatory licensure examination to improve residency application competitiveness, students and their advisors should be aware of the range of possible outcomes.
